# Marketplaces as Public Spaces in Times of The Covid‐19 Coronavirus Outbreak: First Reflections

**DOI:** 10.1111/tesg.12431

**Published:** 2020-06-12

**Authors:** Emil van Eck, Rianne van Melik, Joris Schapendonk

**Affiliations:** ^1^ Institute for Management Research (IMR) Radboud University Nijmegen P.O. Box 9108 6500 HK Nijmegen the Netherlands

**Keywords:** COVID‐19 coronavirus, marketplaces, public spaces, representations, institutional responses, the Netherlands

## Abstract

Marketplaces are regarded as quintessential public spaces, providing not only access to fresh produce but also functioning as important social infrastructures. However, many marketplaces closed down or changed fundamentally in response to the COVID‐19 coronavirus outbreak. In this paper, we reflect on the effects of the crisis on Dutch marketplaces from two interdependent analytical levels. From a ground level, we illustrate their ‘temporary death’ as public spaces and reflect on their changing social dynamics. From an organisational level, we analyse traders’ responses to the institutional measures taken to combat the crisis. Combining pre‐corona, in‐situ research with (social) media analysis, we show how a variegated institutional landscape of market regulation emerged. Whereas some markets closed down, others remained open in a highly regulated manner; representing merely economic infrastructures. Our first reflections lead to new avenues to explore how the COVID‐19 crisis affects the everyday geographies of public space.

## Graphical abstract

Many marketplaces worldwide closed down or changed fundamentally in response to the COVID‐19 coronavirus outbreak. Focusing on Dutch marketplaces, this paper reflects on the variegated institutional landscape of market regulation that emerged during the crisis, offering both opportunities and uncertainties, and how this changed the social dynamics of marketplaces.

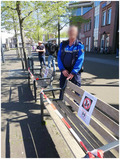

## Introduction

The outbreak of the COVID‐19 coronavirus allegedly started at the Huanan Seafood Wholesale Market in Wuhan, China. Although this particular location of the outbreak is contested, with a number of first coronavirus cases having no proven link to this marketplace (Huang *et al*. 2020), the Wuhan authorities decided to close down the market and ban the trade of live animals at the city’s wet markets on 1 January (Woodward 2020). At these busy and narrow markets, with little possibility for physical separation, traders, customers, and live and dead animals are situated in close proximity to each other, enabling zoonotic diseases to spread from animals to people (Guan *et al*. 2003). Three weeks later, Chinese officials also acknowledged the risk of human‐to‐human transmission and locked down the entire city of Wuhan. During this time, however, international travel continued which marked the start of a pandemic arousing a series of ‘transsystem social ruptures’ (Wachtendorf 2009) that have affected social and economic life in a high variety of ways.

In many regions of the world, marketplaces remained at the centre of attention with regard to preventive corona‐related measures. The dense, open and public character of marketplaces is considered as a major risk of a further uncontrollable spread of infections (Munster *et al*. 2018). At the same time, marketplaces have always been at the heart of urban life, and, despite the rise of commercial retail chains and online shopping, still play a significant role in local food supply and regional economies (Gonzaléz 2019). This explains some of the ambiguity regarding marketplace‐related interventions in Europe. Some markets closed down, whereas others were allowed to remain open. In the Netherlands, regulation appeared to vary to a great extent through time and space, with local authorities making many different judgement calls.

This research is part of a larger international research collaboration which focuses on the mechanisms behind the production of marketplaces as inclusive public spaces with a particular focus on traders’ mobility and place‐making practices.^1^ Like so many aspects of life, our ethnographic work at our main fieldwork locations (two markets in Valkenswaard and Amsterdam) was disrupted by the crisis. As an alternative, we have mainly conducted fieldwork‐from‐a distance to reflect on the ways in which the pandemic has impacted Dutch marketplaces. This consists of the analysis of (social) media coverage and relevant websites of, for example, the national traders association (*Centrale Vereniging voor Ambulante Handel*, CVAH). This paper is based on our last episode of in‐situ research in Valkenswaard, complemented with the above mentioned fieldwork‐from‐a distance on wider developments in the country regarding marketplaces. All quotes in this paper are translated from Dutch to English by the authors.

On 15 March, the Dutch cabinet adopted the first strict measures to slow the spread of the coronavirus throughout the country. Having shunned the stricter measures and complete lockdowns of other European states, the Dutch cabinet chose to pursue an ‘intelligent’ or ‘targeted’ lockdown in the aim to cushion the social and economic costs of complete isolation (Holligan 2020). Whereas all schools, restaurants, cafes and sport clubs were ordered to close their doors, most outdoor public spaces remained open albeit subjected to strict regulations (Rijksoverheid 2020a). They required people to keep a distance of one‐and‐a‐half metres and forbade gatherings of more than 100 people. Adults risked a fine of 390 euro for violating these social distancing rules (Rijksoverheid 2020b).^2^

In this paper, we provide some first reflections on the effects of these crisis measures on marketplaces throughout the Netherlands. By approaching marketplaces from two different, yet mutually constitutive, analytical levels in times of crisis management, we contribute to the study of marketplaces as public spaces in two distinct ways. First, from a ground level (and focusing mainly on our case study Valkenswaard), we observe the sudden changing social infrastructure of marketplaces as vital meeting sites of unfettered social interactions between residents in cities and villages (Klinenberg 2018). This has resulted in, as we put it, a ‘temporary death of public space’, varying from a total absence of public space to changed intensities of its inherent social dynamics. We identify a set of representations of marketplaces that have changed through time to legitimise their closures and controlled re‐openings.

Second, from an organisational level (and including marketplaces throughout the Netherlands), we illustrate that these different measures have resulted in a variegated institutional landscape of marketplace regulation. Although scholars of crisis governance have already shown that formal authorities struggling with crises tend to rely on ad hoc policy measures of ‘command and control’ (e.g. Simo & Bies 2007; Tierney 2012; Boersma *et al*. 2014), we find it remarkable that the crisis responses to the regulation of marketplaces highly varied between local jurisdictions when compared to the consistent, nationally imposed closures of gathering sites in other sectors, such as sport facilities and restaurants. We reflect on how this institutional ambiguity of dispatched, and sometimes contradictory, local crisis measures has affected market traders. Although the inconsistency in regulation allowed for flexibility and alternative trading practices, it also aroused uncertainty among traders. In a direct response to the sudden closure of some marketplaces, they organised several spontaneous protests throughout the country and, in collaboration with the CVAH, collectively claimed their discrimination in the retail market. Our first reflections lead to new avenues to explore salient questions on the everyday geography of public space in times of crisis management that have emerged from our study, as outlined in the conclusion.

## Marketplaces and the Temporary Death of Public Space

Since the beginning of the 1990s, theoretical debates on the social‐political function of public space have proliferated considerably. Having grounded the public sphere in physical space (Low & Smith 2006), scholars from many disciplines ranging from sociology, geography to political science conceived of public space as the site for the formation of democratic culture, political practice and conflict (e.g. Lofland 1989; Carr *et al*. 1993; Mitchell 2003). During the same period, however, the tone of this scholarship became pessimistic and, somewhat paradoxically in the light of the growing debate, widely proclaimed the end or ‘death’ of public space.

Commercialisation and privatisation were identified as the two main processes that transform public space and mark its decline (e.g. Davis 1992; Sorkin 1992; Smith 1996). Both processes are conditioned by, and constitutive of, new urban regeneration strategies of local governments. The consequent ‘sanitising’ of public space is often considered as a moral obligation of local officials to further the goals of neoliberal governance agendas in enforcing corporate and commercial property rules instead of facilitating the voices of the users of space, together with their collective deliberations, social negotiations and political activities (Smith 1998).

González and Waley (2013) have made the cogent argument that the vulnerable position of many traditional retail markets in the Western world can be explained in this context of crumbling public spaces. The waning visitors’ numbers and declining turnover rates of many marketplaces are not merely the consequence of rational customer behaviour but of coordinated state and market strategies that orchestrate covert strategies of retail gentrification. Drawing on the work of the above‐mentioned authors that proclaim the death of public space, González and Waley (2013) conclude that restructuring processes are being facilitated through first rounds of severe disinvestments in markets. These urban revitalisation processes are coupled with an evaluation of markets as asset values instead of public spaces with socio‐political importance where different communities meet each other and benefit from mutual interactions (Janssens & Sezer 2013).

During the corona crisis, many public spaces throughout the world were suddenly closed or subject to strict regulations to restrict the formation of dense public gatherings and the further spread of the virus (Parnell *et al*. 2020). As such, the corona crisis adds a third process producing the ‘death’ of public space. In addition to the privatisation and commercialisation of public spaces, health‐related regulations by local governments impact the nature of public spaces as important meeting places. Within this scenario, however, the ‘temporary death’ of marketplaces came in different forms and intensities. Some were forced to close down through which all social interactions at the heart of their existence ceased to exist. The markets that were allowed to remain open were subject to top‐down imposed regulations that cut back the physical conditions of marketplaces which determine the development of social interactions and negations undergirding the nature of public spaces as social infrastructures (Klinenberg 2018).

In order to analyse how the corona crisis has contributed to the temporary death of public space, we follow Schappo and van Melik’s (2017) distinction in studying marketplaces at two interdependent analytical levels. They distinguish the ‘ground level’ of marketplaces, where social interactions between visitors and traders occur, from a broader ‘organisational level’, where the everyday producers of marketplaces, that is, the traders, interact with many different stakeholders in bringing marketplaces, and their social dynamics on the ground, into existence through their spatial practices. This organisational level encompasses, as such, the support networks, mobility trajectories and organisational strategies of traders that develop beyond the physical limits of marketplaces themselves.

First, with regard to the ground level of public spaces, studies on marketplaces have identified their importance as publicly accessible sites of gatherings with ‘distinctive systems of social relationships’ (Geertz 1978, p. 29; De La Pradelle 2006; Watson 2009). As prototypical public spaces that are constructed, deconstructed and reconstructed on a daily or weekly basis, they function as important spaces for recurring socio‐spatial interactions. From this perspective, marketplaces are vital parts of *social* infrastructures of cities and villages. For Klinenberg (2018), social infrastructures refer to the physical conditions that determine whether social relations and capital develop. When these physical conditions are robust, ‘it fosters contact, mutual support, and collaboration among friends and neighbours … not because they set out to build community, but because when people engage in sustained, recurrent interaction, relationships inevitably grow’ (Klinenberg 2018, p. 5).

Evaluating the physical conditions of marketplaces, Dines (2007) has argued that the particular spatial configurations of marketplaces – ‘the lines of stalls, aisles and series of openings set back from the busy pavements’ (Dines 2007, p. 8) − facilitate the settings for routine and unexpected encounters. In the same vein, De La Pradelle (2006, p. 17) depicts marketplaces as ‘labyrinths of densely crowded, narrow streets and squares’ where the gatherings of different bodies spread out in apparent disorder or ‘contained chaos’ (Black 2012). Watson (2009) characterises the ensuing public performance in marketplaces with the term ‘rubbing along’, through which people who are unknown, or categorically known to each other, develop a feeling of belonging – or ‘public familiarity’ (Blokland & Nast 2014) − in each other’s presence. From this analytical viewpoint, marketplaces can be approached as ‘open regions’ – publicly accessible venues where persons, acquainted or not, ‘have a right to initiate face engagements with each other for the purpose of extending salutations’ (Goffman 1963, p. 132) and the building of ‘mutual appreciation and trust’ (Goffman 1963, p. 84).

As such, fleeting interactions and more durable relationships of care seem to thrive on the open and volatile physical conditions of marketplaces which continuously reoccur through daily or weekly cycles (see also Janssens 2017). Precisely because of these rhythms, as Latham and Layton (2019, p. 3) have argued, the social infrastructure of public spaces are ‘transparent – when being used, infrastructure is not necessarily noticed’ and only ‘becomes visible upon breakdown’ (Star 1999, p. 382). The sudden disruption in the conventionalised social practices in marketplaces in the face of their temporary death, therefore, is likely to impact people’s sense of reflexive awareness and common experience of community and belonging.

Second, with regard to practices of traders at the organisational level, Schappo and Van Melik (2017) have shown that traders produce marketplaces through sustained interactions with stakeholders (including, among others, government officials, other entrepreneurs and members of traders’ associations) that are situated in spatially undifferentiated networks that surround the physical limits of marketplaces. Behind the façade, traders develop broad social networks of support and conflict, as they often do not have permanent workplaces. They navigate themselves spatially and socially between different marketplaces in the region in which they sell and beyond, which is important in underscoring the translocal dimension of both their occupational stature (Peace 1998) and the marketplaces they produce on an everyday basis (Low 2014). Given the fact that some marketplaces closed down, whereas others remained open, it can be expected that traders have to adapt their everyday practices in different ways to sustain their businesses and livelihoods. Moreover, as crisis management scholars have shown, in attempts to cope with, and resist, sudden changes in lifestyle and income during times of crises, people often collectively organise themselves and develop spontaneous initiatives out of shared grievances and solidarity (e.g. Vaiou & Kalandides 2016; Boersma *et al*. 2019). At this broad organisational level, traders not only adapt their everyday practices but also draw on their support networks of varying stakeholders in their aim to realise socio‐institutional change.

In the following sections, we first empirically substantiate the changing social dynamics of public space from the ground level by analysing one of our main fieldwork locations: the weekly market of Valkenswaard. Thereafter, we draw on the organisational level by showing how traders have responded to the variegated institutional landscape of market regulation throughout the whole country.

## Ground Level: Changing Social Dynamics of the Market of Valkenswaard

In the period preceding the outbreak of the coronavirus, the marketplace of Valkenswaard, a small village located in the southern province of North Brabant, had long been celebrated as one the best and busiest weekly markets of the 968 marketplaces in the Netherlands (Municipality of Valkenswaard 2013; CBS 2017). Therefore, the commonly dense and public character of the marketplace of Valkenswaard lends itself particularly well to analyse the effects of the corona‐related preventive measures on the changes of marketplaces as important infrastructures. From this ground level, we illustrate how the sudden closure and re‐opening of the marketplace reflect different stages in the temporary death of public space after local authorities deployed different representations of space to justify their contradictory decisions.

On 17 March, Mayor Ederveen declared an emergency degree to close down the market for at least three weeks. Despite the decision of the overarching safety region of the province to allow weekly markets, the mayor ruled against the decision and deemed the continuation of the village’s usually crowded marketplace ‘irresponsible’. In a written explanation, he wrote:

The current [nationally imposed] conditions – [that of] one‐and‐a‐half metres distancing and no more than 100 people – cannot be maintained in such big markets as that of Valkenswaard. […] Even if we fence‐off the market square with road blocks, that will not be feasible. That is why I stick to my decision. (Liebrand 2020).

After asking the policy‐maker of the municipal department of Economic Affairs about the motivation behind the mayor’s decision, it became clear that, at first instance, the marketplace was considered as a place that acts as a ‘pull factor’ of ‘hazardous’ public gatherings responsible for an uncontrollable spread of the virus. As the policy‐maker explained:

The mayor predicted that the market would attract a lot more visitors than other markets in the region, since the weekly marketplace of Valkenswaard is one the biggest in the region … The marketplace has many narrow streets where people queue up. That is a risk. The mayor expected that it would be impossible for community service officers [BOA’s] to maintain the order of one‐and‐a‐half metres distancing. (Interview April 2020)

Quite suddenly, however, in the midst of this temporary death of the marketplace − during which all socio‐economic interactions came to a standstill for two weeks − the market manager informed all the traders on their collective Facebook‐page that the mayor had revoked his earlier decision to completely close down the market until 2 April, as also reported in a regional newspaper:

The last two weeks, the market has been called off completely. In an amended [spatial] layout, however, the market is allowed to continue… In the case of Valkenswaard, only 24 food pitches will be set up instead of the 75 market pitches that are available during a fully occupied market. The market pitches will be set up in such a way that visitors have enough space to keep a distance from each other. Only food products are allowed for sale. Moreover, people are not allowed to consume their products on the market terrain. (van den Eijnden 2020)

The interviewed policy‐maker explained that the mayor had adopted the advice of the safety region and the Dutch cabinet to allow the continuation of marketplaces which were conceived of as ‘vital parts of the food chain’ (Rijksoverheid 2020b). Also in other marketplaces throughout the country, governmental officials discursively reframed marketplaces as economic asset values by putting them on par with supermarkets. A spokeswoman of the safety region Amsterdam described the market as ‘part of the vital food chain where many residents do their groceries’ (Goedegebuure 2020). Similarly, the market official of Wageningen approached marketplaces as ‘extensions of supermarkets’ and emphasised that markets relieve supermarkets from the hoarding crowds. Going even further, marketplaces were presented as providing healthier solutions: ‘it’s also better that food is sold outdoors and not in the supermarkets, because it is more crowded in there [in supermarkets]’ (Omroep Gelderland 2020). In a similar vein, the market manager of Valkenswaard emphasised that marketplaces are located in the open air. Moreover, as he argued: ‘In supermarkets there are shopping carts which are not disinfected after use all the time. Markets do not have that problem’ (Liebrand 2020).

**Figure 1:**
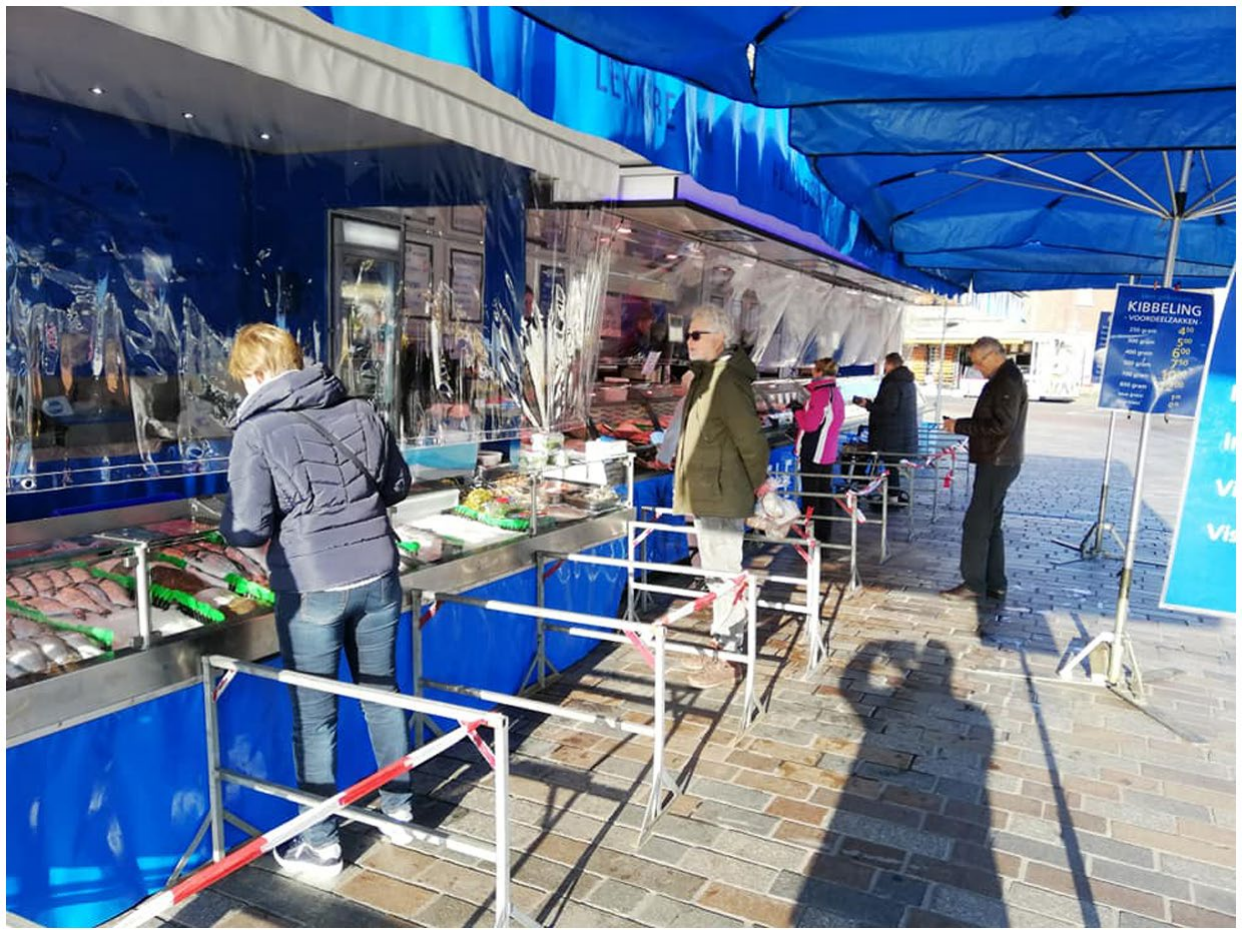
A creative set up of a market pitch to comply with the strict health regulations in Valkenswaard (April 7, 2020). [Colour figure can be viewed at wileyonlinelibrary.com] *Source*: Stichting Weekmarkt Valkenswaard (2020).

**Figure 2:**
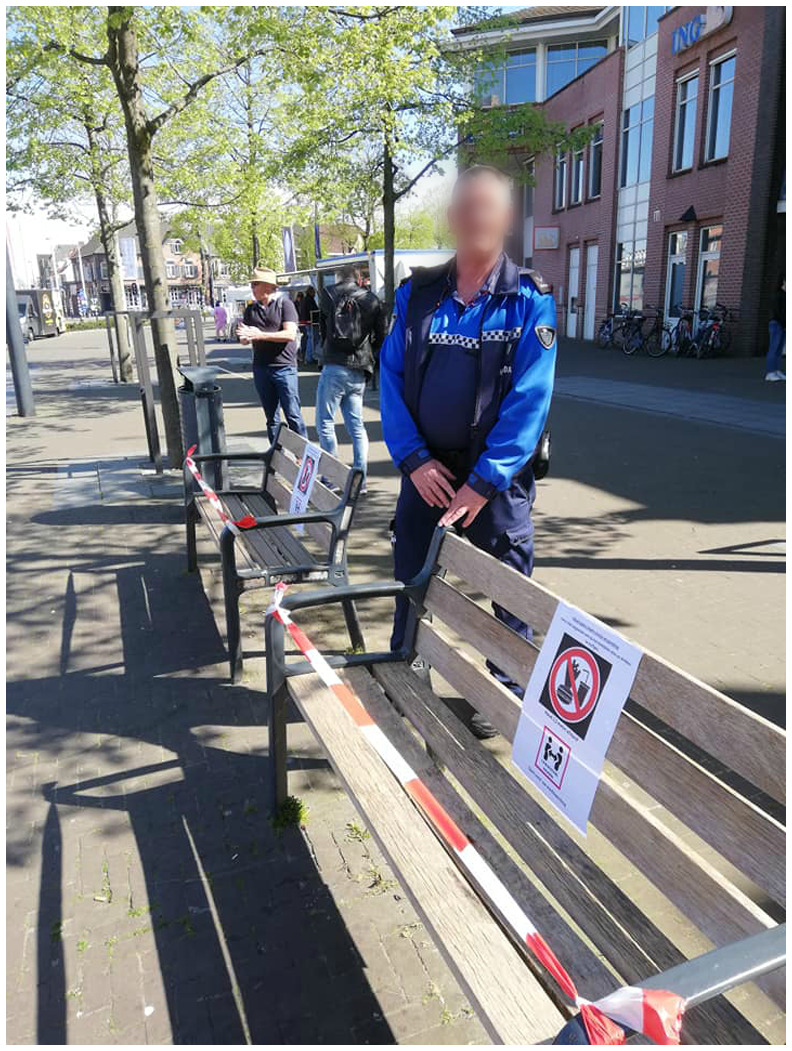
Surveillance and sealed off benches to prevent loitering (21 April 2020). [Colour figure can be viewed at wileyonlinelibrary.com] *Source*: Stichting Weekmarkt Valkenswaard (2020).

As the temporary death of the marketplace gradually gave in under the sway of this new representation of space, the traders were required to comply with the top‐down imposed safety protocols that excluded 51 traders, mainly those in the non‐food sector, from continuing their business. In a response, the traders capitalised upon new possibilities that emerged in the strict regulatory framework. Traders who were allowed to continue their business set up creative constructions to safeguard the health of the visitors and themselves. A butcher placed dividing fences in front of his market pitch to make sure that visitors queue up with a distance of one‐and‐a‐half metres from each other. Just as in supermarkets to protect the cashiers from coughing customers, the butcher also closed off his market pitch with see‐through plastic (Figure 1). Moreover, the market manager placed several posts in front of the market pitches with instructions how to behave in public space. These instructions warn visitors in capital letters to ‘keep distance’ and prohibit them to consume their products on the market terrain. To prevent loitering, standing tables were removed and benches were sealed off with cordon tape (Figure 2).

**Figure 3:**
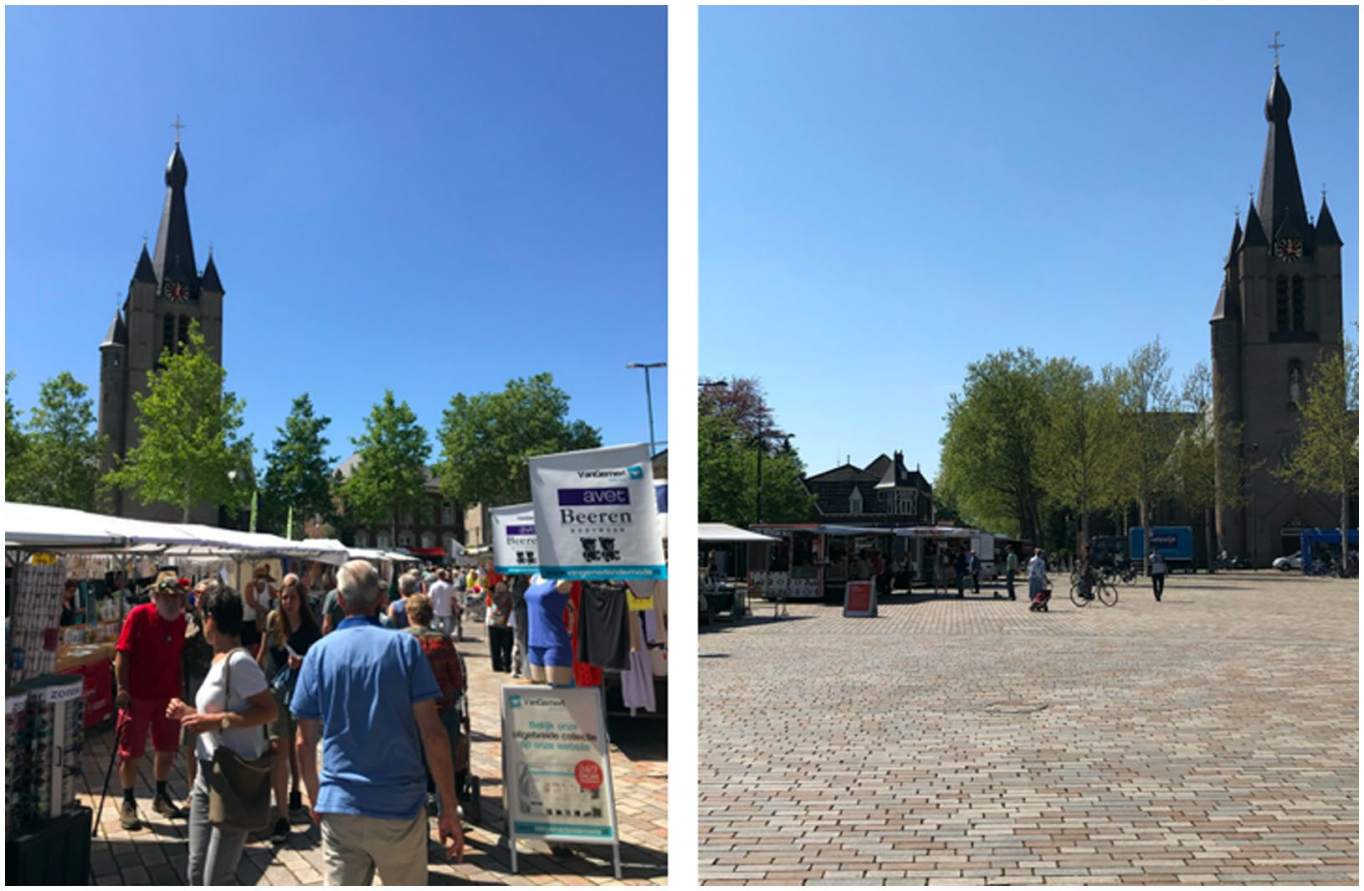
Sunny market days in Valkenswaard before (left, 4 July 2019) and during (right, 23 April 2020) the corona crisis. [Colour figure can be viewed at wileyonlinelibrary.com] *Source*: Emil van Eck.

Although the market functioned again as an important economic selling place of produce after its re‐opening, the social dynamics and its organisation changed. As visitors were no longer allowed to hang out in the open space of the market terrain but only to functionally do their shopping, it suddenly became quiet and orderly (Figure 3). The imposed order on the practices of the market, driven by ideas of safety and control, resembles Smith’s (1998) notion of ‘sanitised’ space or Flusty’s (1997) notion of ‘prickly’ space: both difficult and uncomfortable to occupy or appropriate for alternative uses other than economically motivated. These new safety regulations introduce a dissonance in the pre‐existent, complex social systems of marketplaces (Marovelli 2014), which might explain the concerns of some users of space. We found on the market’s Facebook‐page how a regular visitor recounted his first impression of the new social dynamics of the marketplace:

The smell of roasted chicken floats by and I have the feeling that I am walking on a *real* market again. But I miss the uproar of people, the traders who shout out their prices and the muttering of people who move around. The constantly threatening danger of Corona looms everywhere. Yesterday, here in Valkenswaard, the village where I live, the weekly market continued in a *controlled* manner. The market pitches were far apart, there were fences in front of the pitches and dividing lines were drawn on the ground. Everyone was cautious to not come in each other’s proximity and those unaware of this rule were reprimanded. […] The traders are struggling to sell their wares because of Corona. At least some of them were able to cover the costs, but they were not earning a lot of money and only hoped for a better future where we are freed from Corona. One trader even continued: he hoped that the traders could go on like this next week. (emphasis added).

The visitor conflates a ‘real’ market, characterised by the uproar and muttering of people moving around – that is, fleeting forms of ‘rubbing along’, such as connecting, lingering and taking pleasure in the shared commons (Watson 2009, p. 1589) – with a ‘controlled’ market of separated market pitches and dividing lines where people are constantly conscious of, and on their guard against, unexpected movements and social interactions. This separation is important, as people seem to derive their sense of ‘real’ marketplaces from their easy sociality that either incorporates or challenges the definition of the situation being proposed. As such, the breakdown of the physical conditions of marketplaces that facilitate the chaotic and unpredictable forms of social interactions in the form of open regions (Goffman 1963) indeed reveals, as Latham and Layton (2019) have argued, their transparency in the development of people’s sense of reflexive awareness and common experience of community and belonging.

This does not mean, however, that the economically‐oriented and controlled marketplace completely lost its social value. Precisely because the corona‐related preventive measures render physical interactions less self‐evident and actualising, as geographer Uitermark recently argued in an interview (Ham 2020), it simultaneously highlights people’s mutual interdependencies. In order to help the traders in the non‐food branch who remained unemployed, for example, food‐traders attached white leaflets with contact details of non‐food‐traders to their market pitches. The first sentence on these leaflets read that the non‐food‐traders are ‘unfortunately unable to be present at the market’, but nevertheless accessible for orders and delivery by phone, email and other digital platforms. Moreover, traders and visitors collectively shared the Facebook‐messages of the non‐food‐traders that ask people to stay in touch. For example, a trader of haberdashery posted the message: ‘Out of sight, but not inaccessible! Can we help you? Call, app or mail!’

## Organisational Level: Responses to a Variegated Institutional Landscape

The case of Valkenswaard shows how it was first cancelled and then reopened in a much smaller and more regulated manner. Such dynamics of cancellations and regulations could also be observed elsewhere in the country, albeit in different ways. As noted by Connolly *et al*. (2020), the issue of jurisdictional authority turns out to be noteworthy in the context of public health and its linkage to particular governance relationships that exist between local governments. Crisis governance literature shows that complex social problems, such as those associated with infectious outbreaks, do not fit neatly within the purview of one single jurisdictional authority. Rather, these problems are often addressed through emergent multi‐organisational networks that lack central coordination (Simo & Bies 2007; Tierney 2012; Boersma *et al*. 2014).

Below, we illustrate how this resulted in a variegated institutional landscape of market regulation in the Netherlands. On the one hand, as shown above, the institutional ambiguity enabled some traders to continue their business in the markets that had been allowed to remain open; on the other, it fed collective grievances and emergent protests of those traders operating in markets that had been ordered to close down.

The case of Rotterdam exemplifies this *par excellence*. The municipality of Rotterdam was one of the first cities that decided to temporarily cancel its markets by means of an emergency degree after having received this order of the overarching safety region Rotterdam‐Rijnmond. This decision was fiercely opposed by the Rotterdam traders, who felt being discriminated against as other indoor trading locations, particularly the adjacent food court called the ‘Markthal’, were not summoned to close their doors (Rijnmond 2020a).^3^ In a spontaneous, direct response from the ground that acted as a ‘critical moment’ in challenging the authoritative decisions to close down markets (Verloo 2018), more than 100 traders marched into the Markthal and confronted a municipal official of City Management to express their concerns. Explaining the motivation behind this spontaneous protest, a trader commented:

[By walking into the Markthal], we wanted to direct the attention to the fact that more more than 100 people are continuously present in the indoor space [of the Markthal] and that nobody monitors that. Our outdoor space is set aside, but they [city officials] do nothing with indoor spaces, where the same [types of public gatherings] happen. That is discrimination. (Rijnmond 2020a)

At a later stage, the traders joined the national trade union CVAH, which filed a summary proceeding to convince the judge that this decision to close down the market should be reversed. Eventually, the judge ruled against and stated that the decision of the safety region, as a generally binding declaration, was justified (CVAH 2020a). However, this judgement only applied to the safety region Rotterdam Rijnmond; other markets in the country could continue unless their respective authorities (i.e. the safety regions) also demanded a lockdown.

Accordingly, a variegated market retail landscape emerged in the following weeks. Some cities like Utrecht and Amsterdam, for example, kept their markets open (Westland 2020). At other occasions, just like in Valkenswaard, only the market pitches selling food were allowed to remain open (Goedegebuure 2020). Some bigger markets were closed in the middle of an operating day, as they attracted too many visitors that did not keep enough physical distance, which was the case in The Hague Market (Rubio & De Jonge 2020) and the daily market on Plein ’40‐’45, located in the western district of Amsterdam (AT5 2020). Finally, in Leiden, the weekly market in the city centre was only allowed to continue as long as the market pitches were divided over two market terrains located elsewhere in the city in order to create more space for visitors to keep distance. Directly resisting the decision of the municipality, almost all traders abstained from opening their businesses at these alternative locations. Supporting this protest in Leiden, the so‐called ‘stall placers’ – that is, the employees of the company responsible for setting up the market pitches – joined the protest and refused to cooperate with the municipality (Omroep West 2020). The stall placers enhanced what Juris (2008) has called ‘affective solidarity’, by transforming emotions such as frustration and indignation, caused by the municipal decision to exclude traders from their public space in the city centre, into a sense of solidarity.

Both in Rotterdam and Leiden, traders and their supporting companies proclaimed their ‘right to trade in public space’ (e.g. Crawford 1995; Crossa 2013; Swider 2015) and ensuing ‘discrimination in the retail market’, since the business practices of supermarkets remained relatively unaffected. Here, the local resistances and protests turn out to be geographically pervasive, as they emerged where state powers were directly enacted (Nicholls 2016). At the same time, the resulting in‐group solidarity and collective power merged with the CVAH as an important ‘broker’ (Tarrow & McAdam 2005), enabling the protesting traders to reach beyond the local scale and amplifying their statements on the national level.

Representing the collective voices of traders, the CVAH gained national attention by proclaiming the discrimination of traders in the retail market by emphasising their financial losses in the face of the decisions to suddenly close marketplaces. The CVAH argued that ambulant trade and other forms of retail need to be treated equally: ‘If shops and malls can remain open, the markets can also be held’ (Westland 2020). As such, the executive director of CVAH stated in a radio interview of the national broadcaster NPO1:

In many municipalities, markets have been closed down … We absolutely do not understand these decisions. I do not think that anyone understands these decisions. The current corona crisis is horrible and the national regulations are judicious. But they have to be *proportionate*. Why can shops remain open, while the ambulant trade has to close down? […] We will go the court to complain about this, not because markets have been closed down *per se*, but because of the irrational way in which this has happened. It is completely, utterly, random! (CVAH, 2020b original emphasis)

Here, it becomes clear that the decisions to close down the markets are in themselves not considered as illegitimate, but rather the disproportionate way in which these decisions had been taken; arousing uncertainty for the economic vitality of traders’ businesses. Media coverage especially focused on the vulnerability of markets, quoting traders that face difficulties in these times of corona. For example, a fishmonger from Spakenburg complained about the long‐term financial consequences of the ad hoc decisions of safety regions to close down marketplaces without being notified in advance: ‘Six of the ten marketplaces where I use to trade on a weekly basis have been cancelled. This is going to cost me ten thousand euros’ (Rijnmond 2020b). Corroborating these personal statements, the executive director of CVAH explained that he received hundreds of calls and emails of concerned traders throughout the whole country ‘who had returned from the vegetables and fruit auction halls with their cars fully packed with merchandise worth ten thousand euros for the coming days, before they discovered that the markets were closed down. These are extraordinary financial setbacks.’ (CVAH 2020b).

These empirical illustrations show that most of the protests organised around a collective narrative of discrimination in the retail market and started with actions from the ground as a ‘critical moment’ (Verloo 2018) in directing national attention to the precarious economic situation of traders. The problem of the exclusion from their profession and their ‘right to trade in public space’, which lay behind their collective grievances, led traders not only to develop alternative practices of solidarity at the ground level, like in Valkenswaard, but also to protests to consistently re‐open all marketplaces in the Netherlands.

## Conclusion and Discussion

Marketplaces act as important economic and social infrastructures throughout the world, although many are suffering from governmental disinvestments to enable new rounds of commercialisation and privatisation (Gonzaléz & Waley 2013). The corona crisis has added an additional challenge to the proclaimed death of public space (e.g. Davis 1992; Sorkin 1992; Smith 1996), as many marketplaces either closed down or remained open in a highly regulated and controlled manner under the sway of strict corona‐preventive health regulations.

In this paper, we looked at the impact of the corona crisis on Dutch marketplaces. In the Netherlands, a highly variegated landscape of market regulation emerged through time and space. By analysing this process from the ground level in one of our main fieldwork sites, the marketplace of Valkenswaard, we found that the representation of the market as ‘unsafe’ morphed into a vision that conceived of the market as a vulnerable yet vital part of the food chain to justify its re‐opening. During this process, the marketplace lost its status as a social infrastructure (Klinenberg 2018) and transformed into a ‘sanitised’ (Smith 1998) or ‘prickly’ (Flusty 1997) public space of rational and immediate economic interactions. In addition, the strict regulatory framework that reduced the physical conditions of easy sociality, or ‘rubbing along’ (Watson 2009), also engendered new forms of social interaction and trading, which poignantly lay bare the mutual interdependencies of people in marketplaces as one of the most critical public spaces in cities and villages.

By also drawing on a broader organisational level, we have been able to empirically substantiate how traders responded to the institutional ambiguity of different, and sometimes contradictory, regulations and judgement calls of local authorities in times of crisis governance throughout the country. On the one hand, the variation in institutional arrangements of marketplace regulation in the Netherlands allowed for flexibility and opportunities of some traders to continue their business, as shown in the case of Valkenswaard. On the other hand, this situation also resulted in uncertainty and financial losses of traders. In the face of sudden, rigorous municipal interferences in the functioning of marketplaces (either complete closures, such as in Rotterdam, or in the form of spatial reconfigurations, such as in Leiden), traders organised direct protests out of ‘affective solidarity’ (Juris 2008) to cope with the crisis. Their exclusion from the right to trade in public space led traders to articulate their discrimination in the Dutch retail market.

Given the immediacy of this analysis, our primary goal was to provide first, modest reflections on the effects of the corona crisis on the functioning of marketplaces as public spaces. By specifically drawing on two interdependent analytical levels, we encourage scholars from different disciplines, dealing with topics ranging from the geography and sociology of public space to crisis governance, to engage with some questions that have arisen from our study and could be answered as soon as more time has passed to provide additional perspectives on the corona crisis. Below we provide three of these questions.

First, our study begs the question how traders and the users of space *themselves* have experienced the social constraints and opportunities that emerged during the temporary death of public space of the corona crisis. Our analysis has shown that the *representation* of marketplaces as economic infrastructures contradicts with the lived experiences of the users of marketplaces, or *representational* space, that occur through social interactions and their associated images and symbols (Lefebvre 1991). Through ethnographic accounts, future research could provide more in‐depth insights into how both dimensions of space have affected each other.

One of the interesting adjustments here are the (im)mobility effects: while corona‐related prevention measures have reduced unpredictable activities of sociality and mobility to utility functions of immediate buying on site, especially market traders seem to have sought mobility strategies to continue their businesses and uphold the social value of public spaces for themselves and visitors, by switching to home‐delivering or providing mutual support in online communities, for example. These mobility strategies could analytically be approached as ‘ways of operating’ as they create a certain play in the 'machine of regulations'through different and interfering practices (De Certeau 1988, p. 30).

Second, despite these potential alternative practices, many traditional retail markets throughout Europe suffer from declining turnover rates, especially in the non‐food sector (Gonzaléz 2019). Therefore, it remains uncertain to which extent markets will financially survive after the crisis and how their changed function as important social infrastructures (Klinenberg 2018) will impact the experience of shared communality in cities and villages in the long run – especially for those people who are often marginalised elsewhere (Madanipour 2004).

Finally, with regard to the economic vitality of marketplaces, it still has to become clear to which extent traders’ protests and proclamation of discrimination in the retail market have been successful in receiving permanent institutional recognition and support. Asking (some) of these questions while extending research to other European countries might help to corroborate, or give nuance to, our first research findings.

## Notes

1 Moving Market Places (MMP): Following the Everyday Production of Inclusive Public Spaces is funded by HERA (Humanities in the European Research Area). For more information, see http://heranet.info/projects/public‐spaces‐culture‐and‐integration‐in‐europe/moving‐marketplaces‐mmp‐following‐the‐everyday‐production‐of‐inclusive‐public‐spaces/.

2 These strict rules eventually led to the (temporary) closure of certain popular public spaces, like beaches, forests and parks.

3 This demonstration took place on 14 March, when the corona crisis first emerged in the Netherlands. Soon thereafter, the national government also decided to close down schools, cafes and restaurants, among which the Markthal.
